# ASAP 2: a pipeline and web server to analyze marker gene amplicon sequencing data automatically and consistently

**DOI:** 10.1186/s12859-021-04555-0

**Published:** 2022-01-06

**Authors:** Renmao Tian, Behzad Imanian

**Affiliations:** 1 Institute for Food Safety and Health, Illinois Institute of Technology, Bedford Park, IL 60501 USA; 2 Department of Food Science and Nutrition, Illinois Institute of Technology, Bedford Park, IL 60501 USA

**Keywords:** Marker gene, Amplicon sequencing, 16S rRNA, Pipeline, Web server

## Abstract

**Background:**

Amplicon sequencing of marker genes such as 16S rDNA have been widely used to survey and characterize microbial community. However, the complex data analyses have required many interfering manual steps often leading to inconsistencies in results.

**Results:**

Here, we have developed a pipeline, amplicon sequence analysis pipeline 2 (ASAP 2), to automate and glide through the processes without the usual manual inspections and user’s interference, for instance, in the detection of barcode orientation, selection of high-quality region of reads, and determination of resampling depth and many more. The pipeline integrates all the analytical processes such as importing data, demultiplexing, summarizing read profiles, trimming quality, denoising, removing chimeric sequences and making the feature table among others. The pipeline accepts multiple file formats as input including multiplexed or demultiplexed, paired-end or single-end, barcode inside or outside and raw or intermediate data (e.g. feature table). The outputs include taxonomic classification, alpha/beta diversity, community composition, ordination analysis and statistical tests. ASAP 2 supports merging multiple sequencing runs which helps integrate and compare data from different sources (public databases and collaborators).

**Conclusions:**

Our pipeline minimizes hands-on interference and runs amplicon sequence variant (ASV)-based amplicon sequencing analysis automatically and consistently. Our web server assists researchers that have no access to high performance computer (HPC) or have limited bioinformatics skills. The pipeline and web server can be accessed at https://github.com/tianrenmaogithub/asap2 and https://hts.iit.edu/asap2, respectively.

**Supplementary Information:**

The online version contains supplementary material available at 10.1186/s12859-021-04555-0.

## Background

Marker gene amplicon sequencing is currently an important and mainstream approach to investigate microbial communities [1]. In this approach, first, the total DNA is extracted from non-axenic samples (e.g. environmental samples), and then, through polymerase chain reaction (PCR) with indexed primers, certain genes are targeted, amplified and subsequently subjected to high-throughput sequencing (HTS). Marker genes are usually selected because (a) they are species-specific; and (b) they contain both conserved and variable regions within their sequences. Using classification methods such as Naïve Bayes algorithm [2], the amplified marker gene sequences can be classified to deduce the qualitative and quantitative community composition, providing valuable insights into ecology and evolution of the microbial communities of interest.

One of the most important advantages of amplicon sequencing over traditional approaches is that it is culture-independent, and thus able to detect all members of the community including those that cannot be easily, or at all, cultured, and it does so providing quantitative information about all the members of the microbial community [3, 4]. Researchers in many fields from ecology, biology, environmental and biological conservation to medical science, agriculture and food safety have widely adopted and used amplicon sequencing in their studies. In food industry, 16S rDNA amplicon sequencing has been widely used for detection of food contaminations with pathogenic microorganisms [5, 6]. In environmental studies, multiple genes including 16S rDNA, 18S rDNA [7], ITS [8], dsrA [9], nifH [10] and others have been used to investigate microorganisms in water [11, 12] and soil [13], and their specific ecological functions, for example, in sulfate reduction and nitrogen fixation that contributes to the biogeochemical cycling. One of the most recent applications of amplicon sequencing is in the growing number of studies of human microbiome (e.g. human gut microbiota) in order to discover links between human health or its lack thereof and the changes in the composition and/or relative abundances of microorganisms, both commensal and pathogenic, that inhabit different parts of human bodies [14–16].

Amplicon sequencing also has some limits. It cannot recover all the prokaryotes due to the limitations of the universal primers and their inherently narrower than complete bacterial target range. Eloe-Fadrosh et al. showed that about 10% of environmental microbial sequences cannot be detected using 16S rRNA amplicon sequencing [17]. Also, in contrast to the ability of whole genome shotgun sequencing to detect viruses [18], amplicon sequencing cannot recover viruses from the environment because viruses have no ribosomal RNA genes. Moreover, its resolution is limited to the species level, in comparison to shotgun sequencing, and it cannot be used to discriminate strains of the same species.

The operational taxonomic units (OTUs) were the most commonly used grouping method for the analysis of amplicon sequencing data [19]. In the context of sequences, an OTU is a cluster of reads that differ from each other by less than an arbitrary threshold (e.g. fixed pairwise sequence dissimilarity of 3%). There are multiple methods and tools to generate OTUs from a batch of sequences, including De novo method used in UCLUST [20], UPARSE [21], Swarm [22], and SortMeRNA [23] which generate cluster sequences without references. By contrast, the closed-reference method, as is applied in the first version of QIIME [24], defines OTUs based on a reference database. Early pipelines including Mothur [25], Dotur [26] and QIIME [24] have been developed for the OTU-based analysis.

Although OTU-based analysis can avoid over-estimation of variants caused by sequencing errors, it yields a low resolution (at species and upper levels) and holds back studies exploring microbial communities at a lower level such as strains. Recent advances in sequencing technologies and post-sequencing error correction algorithms have made the single-sequence variant analysis possible. New tools, including DADA2 [27], UNOISE2 [28] and Deblur [29], have been developed to resolve amplicon sequence variants (ASVs) from Illumina-scale amplicon data without defining OTUs. The application of ASV has improved the resolution of the analysis to a single nucleotide level. Furthermore, using the ASV approach facilitates the combination of multiple projects and studies for any sample inclusion and comparison purposes by simply merging the resulting feature tables.

A typical analysis of ASV-based marker gene amplicon sequencing data begins with the input data in the FASTQ file format that contains the gene sequences and the corresponding quality information. Sequencing of multiple samples in a single sequencing run requires unique barcoding or indexing of each sample so that the sequence data for each sample can be correctly identified and separated later on. In other words, multiplexed data will be demultiplexed, an essential process during which reads are assigned to samples based on the barcode sequences. Then, the reads will be trimmed to remove regions that have low quality. The high-quality region will be denoised to correct sequencing errors, and this assures the accuracy of ASV sequences and avoids the overestimation of genetic diversity. A feature table will, then, be generated that contains the information about sequence numbers of all ASVs in the samples. The feature table needs to be normalized to assure the even sample size for all the samples for a reliable comparison. In order to achieve this, the feature table needs to be resampled for the downstream analysis (e.g. in hypothesis testing). Multiple feature tables of projects can be merged then for integration or comparison purposes.

Currently there are available tools implementing ASV-based analysis, such as QIIME 2 [30], DADA 2 [27] and MetaAmp [31]. However, these tools require much human interference at nearly each step. For the demultiplexing, users need to check the orientation of barcodes in the sequence file and the metadata file by reading the files and determine the input as a parameter for the demultiplexing command. For the quality trimming, users are required to select high-quality region based on a quality profile chart, and the instability of quality (especially at 3′ end) can lead to inaccuracy and inconsistency due to human subjectivity. For the resampling step, users need to determine the optimal resampling depth to generate maximal observation of samples and features, and the numbers can be quite different and dependent on each individual’s experience and expertise. The accuracy and consistency of results may be affected by the human decisions at each step of the data analysis. Here, we have developed a pipeline to address these issues in order to analyze amplicon sequence data automatically and consistently.

## Implementation

### Programming and packages

The pipeline was written in Python (version 3.7, http://www.python.org) and R (version 3.5.1, https://www.r-project.org/). QIIME 2 (26) was used for the sequence data analysis with the standard protocol being followed (https://docs.qiime2.org/). Python package subprocess (builtin) was used to run bash commands. Pandas (version 0.25.3) and Numpy (version 1.18.5) were employed to read and write tables. Regular expression search was performed using Re (version 2.2.1). Bio (version 1.77) was used for sequence object handling, and Zipfile to read and write zipped files (e.g. QZA). R package Vegan (version 2.5-6) was used for ecological analysis such as canonical correspondence analysis (CCA), redundancy analysis (RDA) and variable selection analysis.

### Determination of barcode orientation

In order to determine the correct orientation of barcodes in the metadata file and the sequence file for a correct demultiplexing, ASAP 2 searches the barcodes in the metadata against all the reads in the sequence file using grep with regular expression options. The reads that start with a barcode without 5′ insertion and mismatch are considered to have the same orientation while those that start with the reverse complement barcode without 5′ insertion and mismatch are considered to be in reverse complement orientation. Only when the number of reads with one orientation (e.g. the same) is > 10 times more than the other orientation (e.g. reverse complement) does the pipeline determine the barcode orientation as the major one.

### Detection of optimal high-quality region

To eliminate inconsistencies caused by human interference in selecting high-quality region, which is required in QIIME 2 (26) and DADA 2 [27] protocols, ASAP 2 auto selects the optimal high-quality region for the following denoising step in analysis. The pipeline first converts the quality score (median) of each position into a moving average for five nucleotides (n = 5) to reduce the volatility. Regions of consecutive positions with quality scores greater than the specified cutoff are then screened. At last, the longest high-quality region is selected, and the coordinates are set as input parameters for the denoising step.

### Determining the optimal resampling depth

For resampling, a fixed number of reads will be randomly selected from each sample, and samples with numbers of read below that will be removed. Therefore, it is important to select an optimal sampling depth to acquire maximal observations of both samples and features. ASAP 2 optimizes the resampling depth so that it leads to the total sequence number of Max(Ri * Ni). In this formula, R is the number of read in each sample; N is the number of samples left after resampling; and i represents the samples with numbers of read from median to minimum.

## Results and discussion

### Workflow of the pipeline

**Fig. 1:**
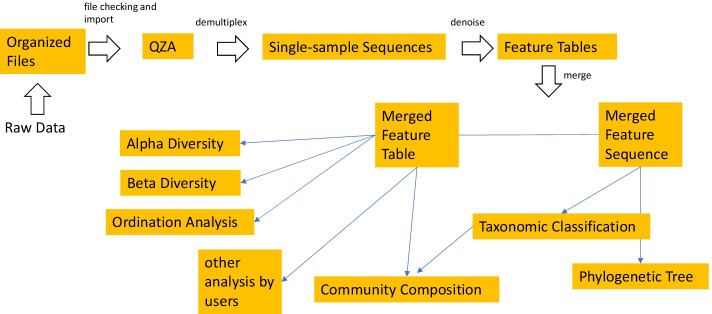
The workflow of the pipeline ASAP 2. The pipeline first imports organized input data as QZA, which are then used for demultiplexing (if applicable). The single-sample sequences are then denoised and feature tables are generated. Multiple projects are then merged to a feature table and a feature sequence file which are used in the downstream analysis. The merged feature table and feature sequence file can be used for other customized analysis. See the detailed file organization, processing and commands used in Additional file 1: Fig. S1

The pipeline starts from sequence data in raw FASTQ or its compressed format (e.g. FASTQ and FASTQ.gz), or feature table with representative sequences (Fig. 1). The sequence files can contain sequences that belong to multiple samples that were barcoded or indexed, pooled and sequenced together (multiplexed) or to only a single sample (demultiplexed). For the multiplexed data, the barcode sequence can be either inside or outside the read files. For all the above FASTQ data files, both single-end and paired-end formats are supported. The pipeline first converts the organized input data to QIIME Zipped Artifacts (qza) format. Multiplexed data will be demultiplexed into single-sample data. For the FASTQ files with barcode data inside or barcode-inside FASTQ files, barcodes will be cut out for demultiplexing with the available option of dual barcode validation. For the FASTQ files with the barcode data in separate FASTQ files, or barcode-outside FASTQ files, barcodes in the metadata file and the sequence file will be compared to determine the orientation for a correct demultiplexing.

Optimal high-quality region of the reads will be identified and selected as an input parameter for the downstream denoising steps through quality trimming, paired ends joining, sequencing error correction, and removing of chimera and PhiX sequences. The high-quality reads will then be summarized to generate a feature table with information about the abundance of ASVs in the samples. The generated feature tables and ASV sequences of multiple projects (if applicable), together with other additional input feature tables and ASV sequences, will be merged for the purpose of combination or comparison.

The merged sequences will be used for phylogenetic tree reconstruction and taxonomic classification. The output of taxonomic classification with the feature table will be used for community composition analysis. Rarefaction will be performed on the merged feature table to eliminate the bias caused by uneven sample sizes in the alpha and beta diversity analysis. A group significance test and a correlation test will be performed to show the effects of environmental factors or treatments on the alpha diversity of samples. Ordination analysis will be performed using the R package Vegan to provide insights about interactions between environmental factors and species in the community.

### Auto detection of optimal high-quality region

**Fig. 2:**
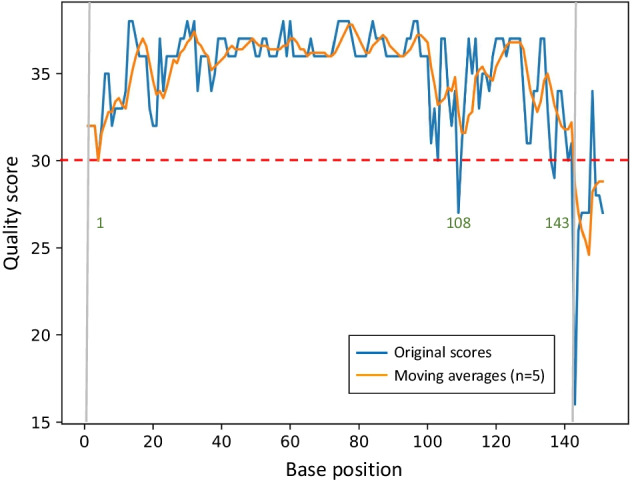
A sequence quality profile demonstrating how ASAP 2 selects the high-quality region for further processing. The original quality score at each position is converted into a moving average score to reduce the volatility caused by occasional drop of score (e.g. at 108) due to sudden quality changes at certain regions. The optimal high-quality region (1–143) is then selected by the pipeline

In order to demonstrate how ASAP 2 detects the optimal high-quality regions, the data set “Atacama soil microbiome” from QIIME 2 documentation tutorial (https://docs.qiime2.org/2020.8/tutorials/atacama-soils/) was analyzed. After the demultiplexing step, the ASAP 2 detected the optimal high-quality region of sequence based on the sequence quality profile. As shown in the sequence quality profile chart (Fig. 2), the original quality score at the position 109 is 27.0 and lower than the cutoff value of 30. However, the moving average (n = 5) at the same position is 32.8 and higher than the cutoff value. Using the moving average quality score instead of original quality score of single bases prevents an undesired and premature truncation at this position and preserves the high-quality bases surrounding the base with an aberrant lower quality. After evaluating the quality of a sequence in this manner, the pipeline selects the longest high-quality region spanning > 50% of the whole sequence length and returns the start and end coordinates (1 and 143) for the quality trimming step.

### Automatic determination of the optimal resampling depth for the rarefaction in the alpha and beta diversity analysis

**Fig. 3:**
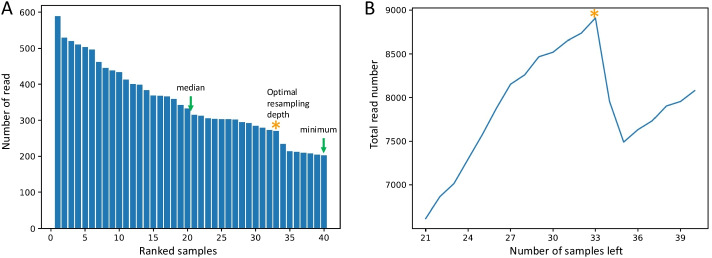
The automatic determination of resampling depth. **A** The profile with numbers of read of the ranked samples shows the selected resampling depth indicated with an asterisk. **B** The total number of read along with the number of samples left shows a peak of total number of read at the selected depth

In the demonstration analysis using the same data set “Atacama soil microbiome” from QIIME 2 documentation tutorial, ASAP 2 determined the optimal resampling depth based on the sample read number profile. For rarefaction resampling, a rarefaction level (resampling depth) was first determined. Samples with the number of reads less than that level were discarded, and the others were rarefied to that level. We determined the rarefaction level by looking at all the samples with number of reads less than the median (so at least half of the samples are retained) and choosing one sample’s read numbers as the rarefaction level which will maximize the total number of retained reads. The median and minimum numbers of reads of the 40 samples were 324 and 202, respectively (Fig. 3A). The 20 samples whose numbers of reads were less than the median were used to determine the resampling depth in order to retain maximal read numbers of all samples post resampling. As a result, there was a peak of total number of read (Fig. 3B) with resampling depth of 270 and 33 samples with 8910 reads.

### Web server interface

**Fig. 4:**
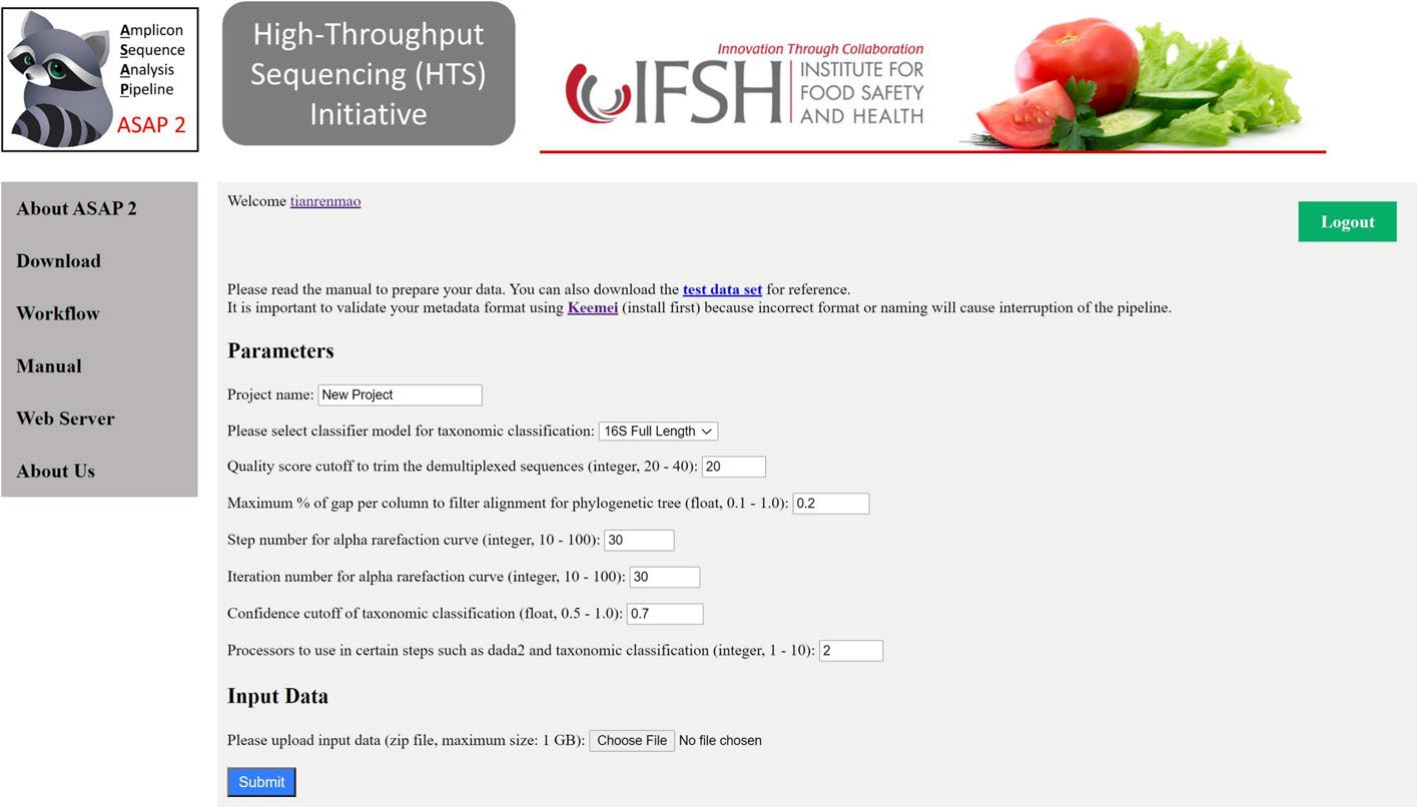
The interface page for job submission on the web server. It allows users to upload their organized data in zip format and set parameters for their task

A web server is also prepared to host ASAP 2 in a high-performance computer (HPC) with 20 cores CPUs (Intel Xeon CPU E5-2660 v3 @ 2.60 GHz, 40 threads) and 512 GB memory. The website (Fig. 4) front end is written in HTML, CSS and JavaScript, and the back end is written in Django. The ASAP 2 website users need to organize their data based on the data formats and submit them with their desired parameters. A typical dataset (100 samples, 1 Gbp) takes about one hour using 10 threads in the web server.

### Demonstration results

**Fig. 5:**
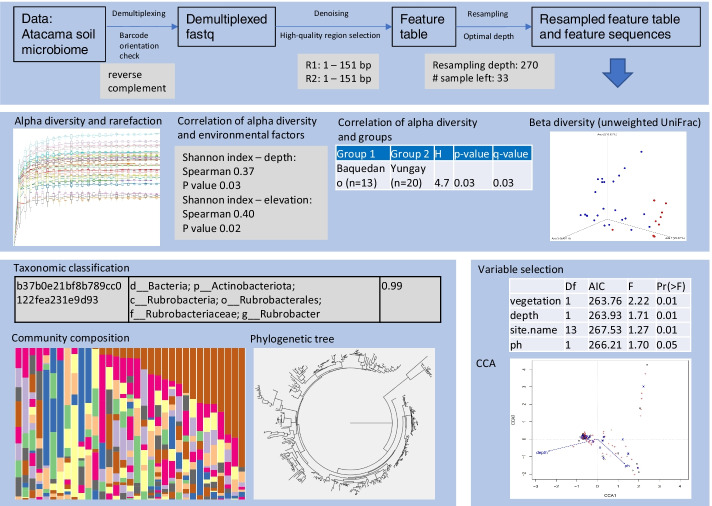
An example of output results generated by the pipeline ASAP 2. The Atacama soil microbiome dataset from QIIME 2 tutorial was analyzed. The results include the alpha diversity in multiple indices and rarefaction, correlation of alpha diversity and environmental factors, correlation of alpha diversity and groups, beta diversity in multiple matrix, taxonomic classification at all taxonomic levels, community composition bar chart, phylogenetic tree, variable selection analysis and CCA / RDA analysis. The result files in QZA or QZV format can be viewed in a web browser using QIIME 2 View (https://view.qiime2.org). In addition, certain features of the results such as sample color are customizable. CCA: Canonical Correspondence Analysis; RDA: Redundancy Analysis

Using the demonstration data set Atacama soil microbiome from QIIME 2 tutorial, ASAP 2 completed the analysis of the organized data and parameter input under no human supervision (Fig. 5). It determined the orientation of the barcode as reverse complement in the metadata file and the barcode sequence file. It selected the optimal high-quality region after demultiplexing, and determined the resampling depth after generating the feature table.

In terms of diversity, the outputs included the alpha diversity indices (number of features, Shannon index, Faith's Phylogenetic Diversity, etc.) and corresponding rarefaction. The rarefaction curves (read counts before resampling) can be used to determine the sequencing saturation that indicates if the sequencing effort is sufficient or not. The effects of environmental factors on the diversity was investigated by correlation analysis. As a result, the depth and elevation were found to have a significant impact on the species diversity. Sample grouping also had an impact on the species diversity and the two groups (different transects) were identified to have a significantly different diversity. Another output was the beta diversity analysis that examines the inter-sample similarities. The result showed that the microbial communities without vegetation were clustered away from those with vegetation (Fig. 5, Beta diversity).

In terms of taxonomy, the representative sequences were classified at all levels. The classification results and the feature table were used to profile the community composition. A phylogenetic tree was constructed using the feature sequences (Fig. 5, Phylogenetic tree), which was also used for the alpha and beta diversity analysis based on phylogenetic distance metrics.

With the community composition profile and the metadata, a variable selection analysis was performed on the same dataset to identify the environmental factors or grouping that have significant impacts on the community composition. Furthermore, a CCA and RDA analysis were used to provide insights into the correlation of environmental factors and taxa. The results showed that vegetation, depth, site and pH are the significant factors affecting the community composition (Fig. 5, CCA), and the species correlating with depth and pH can be observed from the CCA result.

### ASAP 2 in comparison with other pipelines

We made a comparison of ASAP 2 with similar tools QIIME 2, DADA 2 and MetaAmp that perform ASV-based amplicon sequence analysis. In terms of supported data formats, ASAP 2 and QIIME 2 support both multiplexed and demultiplexed sequence data (Table 1), while the other tools only support demultiplexed data. ASAP 2 and QIIME 2 also accept feature tables with representative sequences, and this helps to avoid rerunning the analysis from the beginning.

**Table 1:** Comparison of the pipeline ASAP 2 with other tools in terms of processes that involve manual interference

	QIIME 2	DADA 2	MetaAmp (OTU-based)	MetaAmp ASV-based)	ASAP 2
Feature	ASV	ASV	OTU	ASV	ASV
Supported formats	All *	Only demultiplexed sequences	Only demultiplexed sequences	Only demultiplexed sequences	All*
Barcode orientation identification	Manual	Manual	NA**	NA**	Auto
Combination of multiple projects	Manually merge feature tables	Manually merge feature tables	OTUs cannot be combined	Demultiplexed data as input	Auto merge feature tables
High-quality region identification	Manual	Manual	NA***	Manual	Auto
Resampling depth	Manual	Manual	Manual	Manual	Auto

*Multiplexed/demultiplexed, barcode-inside/barcode-outside, sequence/feature table

**It requires demultiplexed data, so barcode orientation identification is not required

***It does not apply error correction for ASV, so region selection is not required

For multiplexed data, ASAP 2 checks the orientation of barcodes for multiplexed data and reduces errors caused by false input of barcode orientation by users, while other tools require users to check the orientation manually.

Because of the single-nucleotide resolution of ASV in comparison to OTU, ASV-based feature tables and feature sequences from multiple projects can be merged to combine projects for integration or comparison purposes. ASAP 2 can merge multiple projects automatically after generating the feature tables without human interferences while the other tools require a manual merge of feature tables.

It is crucial to select the optimal high-quality region for error correction in ASV-based analysis. However, instability of quality score, especially at the 3′ end where the quality tends to drop, can affect the accuracy and especially consistency of the manual region selection by the users. ASAP 2 yields more accurate and consistent selection of high-quality region in contrast to other tools that involve manual selection based on original quality scores (Table 1).

Also, the inappropriate selection of resampling depth leads to a reduced number of observations of features or samples in the alpha and beta diversity analysis. Unlike the other tools which rely on the users to specify the resampling depth (Table 1), ASAP 2 iterates through all the resampling depth options to determine the optimal resampling depth and yields maximal observation of both samples and features.

## Conclusions

The pipeline runs ASV-based amplicon sequencing analysis automatically and consistently. It supports merging multiple sequencing runs which helps integrate and compare data from different sources (public databases and collaborators). Our web server assists researchers that have no access to high performance computer (HPC) or have limited bioinformatics skills.

The source code of the pipeline is available at https://github.com/tianrenmaogithub/asap2. The web server is available for users to perform the analysis at https://hts.iit.edu/asap2.

## Supplementary Information

**Additional file 1. Fig. S1.** The detailed workflow of the pipeline ASAP 2. The pipeline first imports organized input data as QZA, which are then used for demultiplexing (if applicable). The single-sample sequences are then denoised and feature tables are generated. Multiple projects are then merged to one feature table and a feature sequence file which are used in the downstream analysis. The file naming of each supported data formats are listed at left side, with some examples of QIIME 2 tutorial. The detailed processing and commands used were shown. For the data format codes, fq represents FASTQ; Mu represents multiplexed; De represents demultiplexed; Bi represents barcodeinside; Bo represents barcode-outside; Pe represents paired-end; Se represents single-end.

## Data Availability

The datasets analyzed for the current study are available in the QIIME 2 website (https://docs.qiime2.org/2020.8/tutorials/atacama-soils/).

## Abbreviations

ASAP 2: Amplicon sequence analysis pipeline 2
ASV: Amplicon sequence variant
OTUs: Operational taxonomic units
HPC: High performance computer
PCR: Polymerase chain reaction
HTS: High-throughput sequencing

## Availability and requirements

Project name: ASAP 2. Project home page: https://hts.iit.edu/asap2. Operating system(s): Linux. Programming language: Python 3.6 or higher. Other requirements: R 4.0.5 or higher. License: MIT. Any restrictions to use by non-academics: license needed.
