# Supplementary material for: ASAP 2: a pipeline and web server to analyze marker gene amplicon sequencing data automatically and consistently
Source: BMC Bioinformatics. 2022 Jan 6;23:27. doi: 10.1186/s12859-021-04555-0 (PMC8740450; doi:10.1186/s12859-021-04555-0)
Supplement: Supplementary file 1 — Additional file 1. Fig. S1. The detailed workflow of the pipeline ASAP 2. The pipeline first imports organized input data as QZA, which are then used for demultiplexing (if applicable). The single-sample sequences are then denoised and feature tables are generated. Multiple projects are then merged to one feature table and a feature sequence file which are used in the downstream analysis. The file naming of each supported data formats are listed at left side, with some examples of QIIME 2 tutorial. The detailed processing and commands used were shown. For the data format codes, fq represents FASTQ; Mu represents multiplexed; De represents demultiplexed; Bi represents barcodeinside; Bo represents barcode-outside; Pe represents paired-end; Se represents single-end. [file 12859_2021_4555_MOESM1_ESM.docx]

**Supplementary Materials**

**
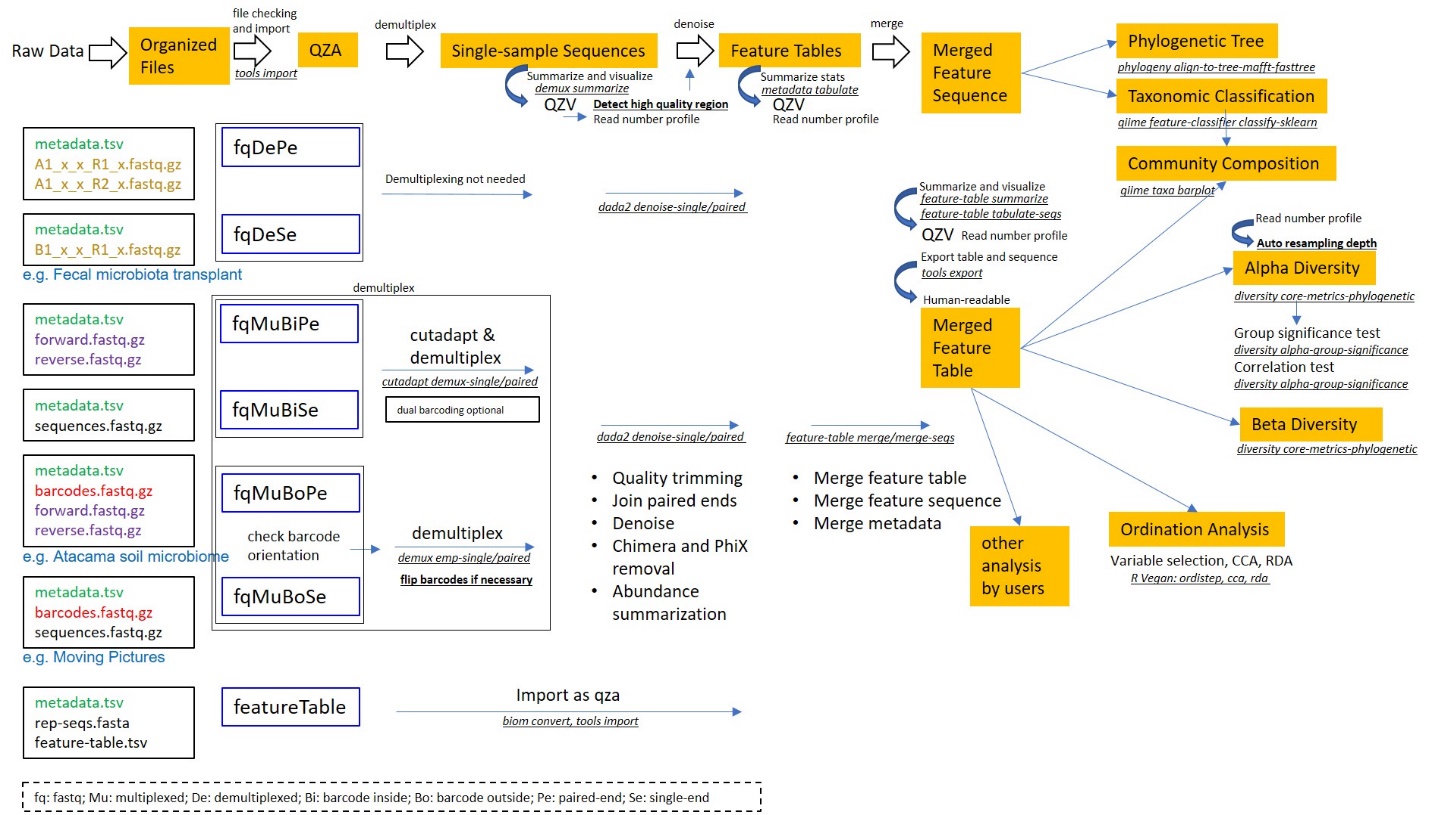
**

**Figure S1.** The detailed workflow of the pipeline ASAP 2. The pipeline first imports organized input data as QZA, which are then used for demultiplexing (if applicable). The single-sample sequences are then denoised and feature tables are generated. Multiple projects are then merged to one feature table and a feature sequence file which are used in the downstream analysis. The file naming of each supported data formats are listed at left side, with some examples of QIIME 2 tutorial. The detailed processing and commands used were shown. For the data format codes, fq represents FASTQ; Mu represents multiplexed; De represents demultiplexed; Bi represents barcode-inside; Bo represents barcode-outside; Pe represents paired-end; Se represents single-end.
